# Systematic Review and Meta-Analysis of the Epidemiology of Vancomycin-Intermediate and Heterogeneous Vancomycin-Intermediate *Staphylococcus aureus* Isolates

**DOI:** 10.1371/journal.pone.0136082

**Published:** 2015-08-19

**Authors:** Shanshan Zhang, Xiaoxi Sun, Wenjiao Chang, Yuanyuan Dai, Xiaoling Ma

**Affiliations:** 1 School of Medicine, Shandong University, Ji’nan, 250061, PR China; 2 Department of Clinical Laboratory, Affiliated Provincial Hospital of Anhui Medical University, Hefei, 230001, PR China; Rockefeller University, UNITED STATES

## Abstract

**Background:**

Vancomycin-intermediate *Staphylococcus aureus* (VISA) and heterogeneous VISA (hVISA) are associated with vancomycin treatment failure, and are becoming an increasing public health problem. Therefore, we undertook this study of 91 published studies and made subgroup comparisons of hVISA/VISA incidence in different study years, locations, and types of clinical samples. We also analyzed the genetic backgrounds of these strains.

**Methods:**

A systematic literature review of relevant articles published in PubMed and EMBASE from January 1997 to August 2014 was conducted. We selected and assessed journal articles reporting the prevalence rates of hVISA/VISA.

**Results:**

The pooled prevalence of hVISA was 6.05% in 99,042 methicillin-resistant *S*. *aureus* (MRSA) strains and that of VISA was 3.01% in 68,792 MRSA strains. The prevalence of hVISA was 4.68% before 2006, 5.38% in 2006–2009, and 7.01% in 2010–2014. VISA prevalence was 2.05%, 2.63%, and 7.93%, respectively. In a subgroup analysis of different isolation locations, the prevalence of hVISA strains was 6.81% in Asia and 5.60% in Europe/America, and that of VISA was 3.42% and 2.75%, respectively. The frequencies of hVISA isolated from blood culture samples and from all clinical samples were 9.81% and 4.68%, respectively, and those of VISA were 2.00% and 3.07%, respectively. The most prevalent genotype was staphylococcal cassette chromosome *mec* (SCC*mec*) II, which accounted for 48.16% and 37.74% of hVISA and VISA, respectively. Sequence Type (ST) 239 was most prevalent.

**Conclusion:**

The prevalence of hVISA/VISA has been increasing in recent years, but has been grossly underestimated. Its incidence is higher in Asia than in Europe/America. hVISA is isolated from blood culture samples more often than from other samples. These strains are highly prevalent in epidemic MRSA strains. This study clarifies the epidemiology of hVISA/VISA and indicates that the detection of these strains and the control of nosocomial infections must be strengthened.

## Introduction

*Staphylococcus aureus*, one of the major nosocomial and community-acquired pathogens, causes a variety of clinical problems, including infections of the skin and soft tissues [1]. Since the 1960s, the prevalence of methicillin-resistant *Staphylococcus aureus* (MRSA) has increased at a dramatic rate [2, 3], and is associated with higher rates of morbidity and mortality than methicillin-susceptible *S*. *aureus* (MSSA) [4].

Glycopeptides, such as vancomycin, are popular and effective antimicrobial drugs for treating MRSA. Unfortunately, vancomycin-intermediate *S*. *aureus* (VISA) and heterogeneous VISA (hVISA) have been reported since 1997. hVISA is an *S*. *aureus* isolate with a minimum inhibitory concentration (MIC) for vancomycin within the susceptible range when tested with routine methods, but in which a proportion of the cell population is within the vancomycin-intermediate range [5]. hVISA/VISA infections are commonly associated with persistent infections, prolonged bacteremia, and/or prolonged hospitalization [6–9]. Today, there is growing concern that hVISA and VISA are becoming prevalent worldwide [10–12].

In recent years, there have been many reports from single medical centers or individual countries of the incidence of hVISA/VISA, but few systematic reviews or meta-analyses on their prevalence. The review by Liu et al. on the epidemiology of hVISA/VISA was published over 10 years ago [13]. Another meta-analysis, by Van Hal et al., selectively analyzed the clinical significance and outcomes of hVISA [9]. In this systematic review and meta-analysis, we pooled the published studies that have reported the prevalence of hVISA/VISA, and made subgroup comparisons of the incidence of hVISA/VISA in different years, locations, and types of clinical samples. We also analyzed the genetic backgrounds of these strains. The results of this study will help to clarify the epidemiology of hVISA/VISA and will advance the control and management of these drug-resistant isolates.

## Methods

### Search strategy

Two independent examiners (S.S.Z. and X.X.S.) performed a systematic literature review of potentially relevant studies pertaining to VISA and hVISA. The studies were identified in the PubMed and EMBASE databases from articles published between January 1997 and August 2014. The following terms and connectors were used in the search strategy: (1) ‘vancomycin-intermediate *Staphylococcus aureus*’, VISA; (2) ‘heterogeneous vancomycin-intermediate *Staphylococcus aureus*’, hVISA; (3) ‘*Staphylococcus aureus* with reduced vancomycin susceptibility’, SA-RVS; (4) ‘glycopeptide-intermediate *Staphylococcus aureus*’, GISA; and (5) ‘heterogeneous glycopeptide-intermediate *Staphylococcus aureus*’, hGISA. The search was restricted to human studies.

### Selection of studies

Studies identified in the literature search were checked by title and abstract. The papers with relevant abstracts were examined in full. The criteria for the inclusion and exclusion of the studies were established by the investigators before the literature was reviewed. The inclusion criteria were as follows: (1) studies that were original articles, short communications, correspondence, or letters that provided sufficient original data about the prevalence of hVISA/VISA; (2) studies in which all MRSA strains were randomly selected; (3) studies that used normative and publicly accepted detection methods for hVISA/VISA; and (4) studies that were published in English. The exclusion criteria were: (1) studies that contained duplicate data or were overlapping articles; (2) reviews and conference abstracts; and (3) articles that included fewer than 10 cases.

### Data extraction

Two authors independently ascertained the characteristics of each study, including the first author’s surname, year of publication, continent, country, study years, isolate source, detection method, hVISA frequency, VISA frequency, and genotypes. When there was disagreement, the relevant paper was reviewed and the differences were resolved by consensus.

### Assessment of study quality

The studies were assessed for quality, and only high-quality studies were included in the analysis. The criteria for high-quality studies were (1) that they provided basic data that included the study period and area, total number of isolates tested, and number of hVISA/VISA isolated; and (2) that they used dilution methods or E-test to detect VISA, and population analysis profile–area under the curve (PAP-AUC), macromethod Etest (MET), or screening agar to detect hVISA. When two studies overlapped, the more recent and larger study was included in the analysis. If one article included more than one study period, it was divided into several independent studies.

### Statistical analysis

Statistical analyses were performed with STATA version 12.0. The data were pooled using the fixed-effects model (FEM) [14] and the random-effects model (REM) [15]. Statistical heterogeneity was assessed using the Cochran Q and I^2^ statistical methods [16]. P < 0.1 was considered statistically significant. For all analyses, the results of FEM are presented only when there was no heterogeneity between the studies. Otherwise, the results of REM are presented. Freeman–Tukey arcsine transformations were performed to stabilize the variances, and after the meta-analysis, we transformed the summary estimates and the confidence interval (CI) boundaries back to proportions using the sine function [17].

## Results

### Results of the systematic literature search

**Fig 1:**
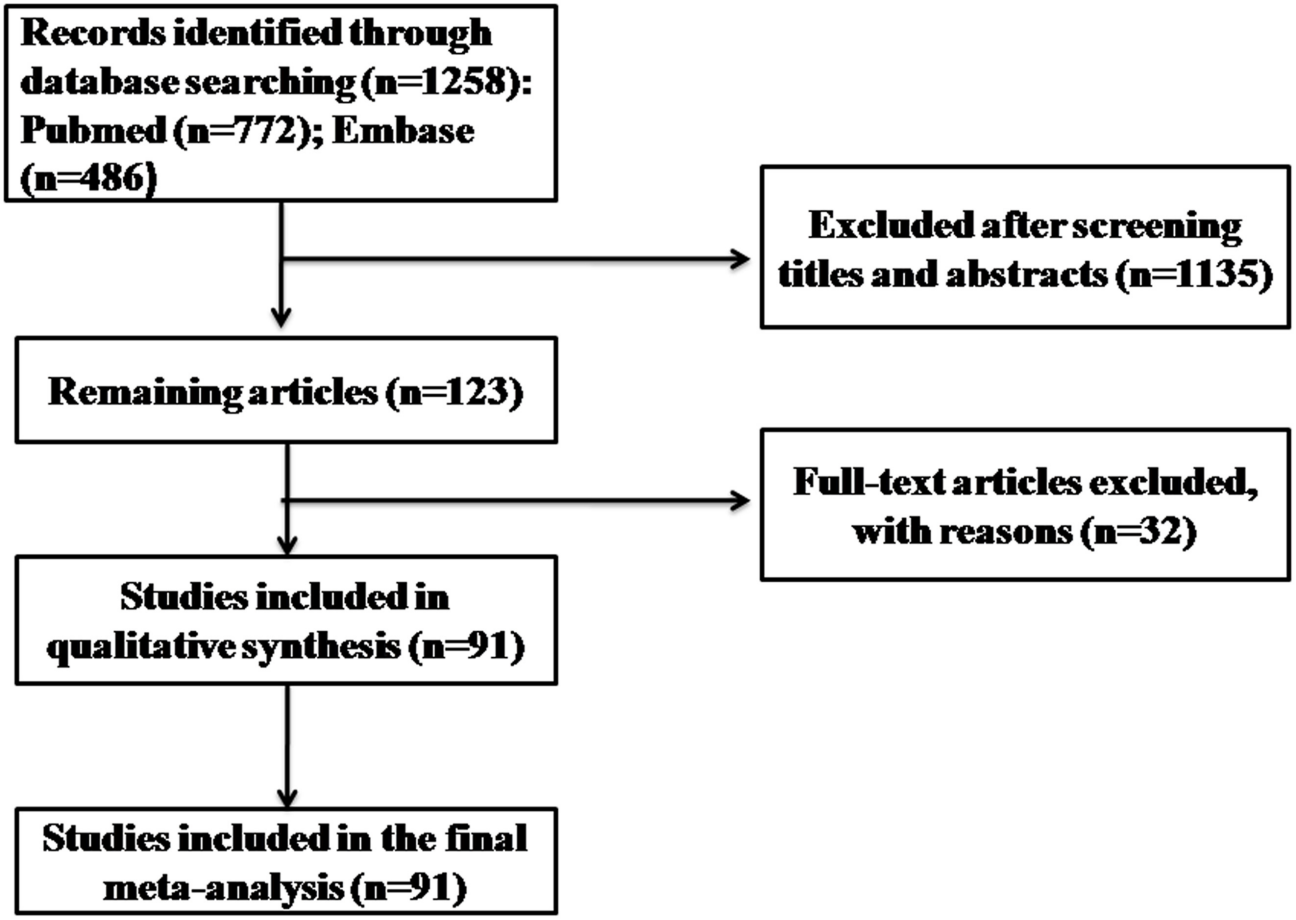
Flowchart of study selection.

In total, 1258 citations were identified in the initial electronic database search. Ultimately, 91 studies were included, based on the inclusion and exclusion criteria (Fig 1). These 91 studies that reported the prevalence of hVISA/VISA included 39 from Asia, 28 from Europe, 21 from America, and 3 from Australia (Table 1) [18–108]. In the pooled analysis, hVISA was reported in 76 studies, with an overall prevalence of 6.05% among 99,042 MRSA strains (95% CI 4.78–7.48), and VISA was reported in 38 studies, with a prevalence of 3.01% among 68,792 MRSA strains (95% CI 1.62–4.83) (Table 2).

**Table 1:** Characteristics of the eligible studies.

Study, Year Published	Country, Continent	Study Year	Isolate Source	Detection Method^a^	hVISA Frequency (%) and Genotype (%)	VISA Frequency (%) and Genotype (%)
Hanaki et al, 2007[76]	Japan, Asia	1978–2005	All clinical samples	E-test		5/2446
						(0.2)5/5 (100) SCC*mec* II
Hiramatsu et al, 1997[36]	Japan, Asia	1996/01–1997/03	All clinical samples	BHI	34/1149 (3.0)	
Song et al, 2004[38]	Asia	1997/01–2000/03	All clinical samples	BHI	58/1357 (4.3)	
				PAP		
Wong et al, 1999[20]	Hong Kong, Asia	1997/07–1998/06	All clinical samples	E-test	3/52 (5.8)	
Ike et al, 2001[75]	Japan, Asia	1997/09–1997/12	All clinical samples	BHI	0/6625 (0)	
				PAP		
Trakulsomboon et al, 2001[45]	Thailand, Asia	1998–1999	All clinical samples	BHI	5/155 (3.2)	
				PAP		
Neoh et al, 2007[56]	Japan, Asia	1998/01–2005/10	Blood samples	PAP-AUC	2/20 (10.0)	
Kim et al, 2002[27]	Korea, Asia	1998/12–1999/08	All clinical samples	BHI	24/3363 (0.7)	0/3363 (0)
				PAP		
Aminaka et al, 2009[34]	Japan, Asia	1999	All clinical samples	BHI	7/138 (5.1)	0/138 (0)
				PAP		
Kim et al, 2000[108]	Korea,Asia	1999/01–1999/08	All clinical samples	PAP	59/3371 (1.8)	
Kim et al, 2003[73]	Korea, Asia	1999/06–2001/01	All clinical samples	BHI	0/439 (0)	
				PAP		
Hsueh et al, 2010[26]	Taiwan, Asia	2001/09–2002/08	All clinical samples	MIC based		43/1500
						(2.9) 43/43 (100) SCC*mec* III
Wang et al, 2009[70]	Taiwan, Asia	2001–2003	All clinical samples	BHI	2/13 (15.3)	8/13 (61.5)
				PAP	1/2 (50.0) SCC*mec* IV-ST59	5/8 (62.5) SCCmec IV-ST59
					1/2 (50.0) SCC*mec* III-ST239	3/8 (37.5) SCCmec III-ST239
Ghung et al, 2010[74]	Korea, Asia	2001–2006	All clinical samples	MIC based	18/41639 (0.04)	15/41639 (0.04)
				PAP-AUC	12/18 (66.7) SCC*mec* II-ST5	12/15(80.0) SCC*mec* III-ST239
					4/18 (22.2) SCC*mec* IV-ST72	2/15 (13.3) SCC*mec* II-ST5
					1/18 (5.6) SCC*mec* III-ST239	1/15 (6.7) SCC*mec* IV-ST72
					1/18 (5.6) SCC*mec* IV-ST1	
Lulitanond et al, 2009[46]	Thailand, Asia	2002/08–2003/04	All clinical samples	BHI	4/533 (0.8)	
				PAP	4/4 (100) SCC*mec* III-ST239	
Maor et al, 2009[25]	Israel, Asia	2003–2006	Blood samples	MET	27/223 (12.1)	
Maor et al, 2007[82]	Israel, Asia	2003/01–2004/12	Blood samples	MET	16/264 (6.0)	
Ho et al, 2010[81]	Taiwan, Asia	2003/03–2003/08	All clinical samples	BHI	7/1000 (0.7)	2/1000 (0.2)
				PAP-AUC		
Aminaka et al, 2009[34]	Japan, Asia	2005–2006	All clinical samples	BHI	3/477 (0.6)	0/477 (0)
				PAP		
Sun et al, 2009[83]	China, Asia	2005–2007	Blood samples	MET	26/200 (13.1)	1/200 (0.5)
				PAP-AUC	20/26(77.0) SCC*mec* III-ST239	1/1 (100) SCC*mec* II-ST5
					5/26 (19.2) SCC*mec* II-ST5	
					1/26 (3.8) SCC*mec* IV-ST59	
Campanile et al, 2010[52]	India, Asia	2005–2007	All clinical samples	BHI, MET	36/139 (25.9)	0/139 (0)
				PAP-AUC	3/36 (8.3) ST8	
					3/36 (8.3) ST239	
					15/36 (41.7) ST247	
					12/36 (33.3) ST228	
					3/36 (8.3) others	
Chen et al, 2011[58]	China, Asia	2005–2008	All clinical samples	PAP-AUC	62/559 (11.1)	0/559 (0)
Fong et al, 2009[23]	Singapore, Asia	2005/01–2006/12	Blood samples	MET	3/56 (5.4)	
Wang et al, 2013[55]	Taiwan, Asia	2005/01–2009/12	Blood samples	E-test GRD	16/284 (5.6)	
				PAP-AUC	7/16 (43.8) ST239	
					5/16 (31.4) ST5	
					1/16 (6.2) ST59, ST45, ST398, ST900	
El Ayoubi et al, 2014[48]	Lebanon, Asia	2006/02–2013/03	All clinical samples	MIC based		5/113 (3.8)
Lulitanond et al, 2009[46]	Thailand, Asia	2006/09–2007/12	All clinical samples	BHI	8/361 (2.2)	3/361 (0.8)
				PAP	8/8 (100) SCC*mec* III-ST239	2/3 (66.7) SCC*mec* III-ST239
						1/3 (33.3) SCC*mec* II-ST5
Wang et al, 2013[84]	China, Asia	2007/07-2009/03	All clinical samples	MET	25/122 (20.5)	
					23/25 (92.0) SCC*mec* III	
					2/25 (8.0) SCC*mec* II	
Hanaki et al, 2014[19]	Japan, Asia	2008/01–2011/05	Blood samples	MET	55/830 (6.5)	8/830 (1.0)
				PAP-AUC		
Park et al, 2012[28]	Korea,Asia	2008/08–2010/09	Blood samples	E-test	101/268 (37.7)	
				PAP-AUC	73/268 (72.3) SCC*mec* II-ST5	
					17/268(16.8) SCC*mec* IV-ST72	
					9/268 (8.9) SCC*mec* III-ST239	
					2/268 (3.0) others	
Gowrishankar et al, 2013[40]	India, Asia	2009–2010	All clinical samples	MHA		10/63 (15.9)
Norazah et al, 2012[79]	Malaysia, Asia	2009/01–2009/12	All clinical samples	E-test GRD	2/43 (4.7)	
				PAP-AUC		
Ramli et al, 2012[94]	Malaysia, Asia	2009/02–2009/05	All clinical samples	E-test GRD	7/320 (2.2)	
				PAP-AUC		
Lin et al, 2012[72]	Taiwan, Asia	2009/03–2009/12	Blood samples	MET	5/62 (8.1)	
				PAP-AUC	3/5 (60.0) SCC*mec* III-ST239	
					1/5 (20.0) SCC*mec* III-ST900	
					1/5 (20.0) SCC*mec* II-ST5	
Dubey et al, 2013[99]	India, Asia	2009/09–2012/04	All clinical samples	E-test		545/1214 (44.9)
Khanal et al, 2010[87]	Nepal, Asia	2010	All clinical sample	Arg screening		80/300 (26.6)
Chaudhari et al, 2015[51]	India, Asia	2010/09–2013/03	All clinical samples	BHI	4/58 (6.9)	
				PAP-AUC		
Panomket et al, 2014[68]	Thailand, Asia	2010/11–2011/11	All clinical samples	BHI	2/68 (2.9)	
				PAP-AUC		
Liu et al, 2014[71]	China, Asia	2011/06–2012/05	All clinical samples	PAP-AUC	17/77 (22.1)	
					15/17(88.2) SCC*mec* III-ST239	
					1/17 (5.9) SCC*mec* III-ST5	
					1/17 (5.9) SCC*mec* II-ST1301	
Kaleem et al, 2012[31]	Pakistan, Asia	2012	All clinical samples	E-test	6/347 (1.7)	
Guo et al, 2013[100]	China, Asia	2012/06–2012/12	All clinical sample	MIC based		1/1790 (0.06)
Chaudhary et al, 2013[85]	India, Asia	2013	All clinical samples	MHA	8/130 (6.1)	
				E-test		
Wootton et al, 2001[69]	UK, Europe	1983–1999	All clinical sample	E-test	0/100 (0)	
				PAP-AUC		
Robert et al, 2006[32]	France, Europe	1983–2001	All clinical samples	E-test		1/1445 (0.07)
Geisel et al, 1999[39]	Germany, Europe	1992–1998	All clinical samples	BHI	7/85 (8.2)	
Kantzanou et al,1999[91]	Greece, Europe	1994–1997	All clinical samples	E-test	1/72 (1.5)	
				PAP		
Uçkay et al, 2012[54]	Switzerland, Europe	1995/01–2003/08	All clinical samples	BHI		55/208 (26.4)
Bierbaum et al, 1999[80]	Germany, Europe	1997	All clinical samples	BHI	2/367 (0.5)	
				PAP		
Bert et al, 2003[88]	France, Europe	1997/01–2002/01	All clinical samples	MET	13/48 (27.1)	
				PAP		
Schmitz et al, 1999[101]	Europe	1997/04–1998/04	All clinical samples	E-test	0/302 (0)	
Marchese et al, 2000[50]	Italy, Europe	1997/08–1998/12	All clinical samples	BHI	2/179 (1.1)	
				PAP		
Canton et al, 1999[90]	Spain, Europe	1997/10–1998/01	All clinical samples	E-test		12/248 (4.8)
Fitzgibbon et al, 2007[60]	Ireland, Europe	1998–2004	All clinical samples	MET	73/3189 (2.3)	
				PAP-AUC		
Sancak et al, 2005[67]	Turkey, Europe	1998/01–2002/01	All clinical samples	MET	46/256 (18.0)	0/256 (0)
				PAP		
Aucken et al, 2000[102]	UK, Europe	1998/05–1999/04	All clinical samples	MIC	0/11242 (0)	
				BHI		
Reverdy et al, 2001[59]	French, Europe	1998/11–1999/04	All clinical samples	MET	5/171 (2.9)	
				PAP		
Lassence et al, 2006[30]	France, Europe	1999–2000	All clinical samples	MHA	11/329 (3.3)	
				E-test		
Denis et al, 2002[41]	Belgium, Europe	1999/01–1999/12	Blood samples	BHI	4/2145 (0.1)	3/2145 (0.1)
				PAP		
Vaudaux et al, 2012[86]	Switzerland, Europe	2000–2008	All clinical samples	MHA		13/57 (31.7)
						13/13 (100) SCC*mec* I-ST228
Nonhoff et al, 2005[65]	Belgium, Europe	2001/01–2001/12	All clinical samples	E-test	3/455 (0.7)	
					2/3 (66.7) SCC*mec* I	
					1/3 (33.3) SCC*mec* IV	
Cartolano et al, 2004[66]	France, Europe	2000/06	All clinical samples	MHA		31/1070 (2.9)
				PAP-AUC		
Garnier et al, 2006[18]	France, Europe	2001/07–2002/06	All clinical samples	MET	255/2300 (11.1)	
				PAP-AUC		
Nakipoglu et al, 2005[61]	Turkey, Europe	2001/09–2002/04	All clinical samples	BHI	7/135 (5.1)	
				PAP		
Mlynarczyk et al, 2003[96]	Poland, Europe	2002	All clinical samples	PAP-AUC	5/103 (4.8)	0/103 (0)
Bataineh et al, 2006[93]	Spain, Europe	2002/04–2004/08	All clinical samples	MHA	5/139 (3.6)	
				E-test		
Piérard et al, 2004[95]	Belgium, Europe	2003	All clinical samples	MET	5/1002 (0.5)	1/1002 (0.1)
				PAP		
Kirby et al, 2010[98]	UK, Europe	2004–2006	All clinical samples	MET	86/2550 (3.4)	
				PAP-AUC		
Lewis et al, 2009[43]	UK, Europe	2005–2007	Blood samples	MET	35/195 (18.0)	
					35/35 (100) SCC*mec* IV	
Parer et al, 2012[78]	France, Europe	2007	All clinical samples	MHA	12/20 (60.0)	
				PAP-AUC		
Sancak et al, 2013[103]	Turkey, Europe	2009–2010	Blood samples	MET	24/175 (13.7)	0/175 (0)
				PAP-AUC		
Rybak et al, 2008[21]	USA, America	1986–1993	All clinical samples	MET	5/225 (2.2)	1/225 (0.4)
				PAP-AUC	3/5 (56.9) SCC*mec* II	
					2/5 (38.4) SCC*mec* IV	
Ariza et al, 1999[104]	USA, America	1990/01–1997/12	All clinical samples	E-test	14/19 (73.7)	
				PAP		
Rybak et al, 2008[21]	USA, America	1994–2002	All clinical samples	MET	27/356 (7.6)	8/356 (2.3)
				PAP-AUC	15/27 (56.9) SCC*mec* II	
					10/27 (38.4) SCC*mec* IV	
					2/27 (4.7) others	
Adam et al, 2010[33]	Canada, America	1995–2006	All clinical samples	E-test GRD	25/475 (5.3)	
				PAP-AUC	16/25 (64.0) SCC*mec* II	
					6/25 (24.0) SCC*mec* I	
					2/25 (8.0) SCC*mec* III	
					1/25 (4.0) SCC*mec* IV	
Musta et al, 2009[105]	USA, America	1996–1997	Blood samples	MHA	8/61 (13.1)	
				E-test	7/8 (93.0) SCC*mec* II	
					1/8 (7.0) SCC*mec* IV	
Hubert et al, 1999[49]	USA, America	1997	All clinical samples	MHA		4/630 (0.6)
				PAP-AUC		
Tallent et al, 2002[106]	USA, America	1997/01–2000/12	Blood samples	MIC based	1/619 (0.2)	
				PAP		
Franchi et al, 1999[97]	USA, America	1997/03–1997/05	All clinical samples	E-test PAP		0/30 (0)
Fridkin et al, 2003[42]	USA, America	1999/03–2000/12	All clinical samples	BHI		6/102 (5.8)
Eguia et al, 2005[63]	USA, America	1999/12–2000/08	All clinical samples	BHI	0/211 (0)	
Musta et al, 2009[105]	USA, America	2000–2001	Blood samples	MHA	5/55 (9.1)	
				E-test	5/5 (100) SCC*mec* II	
Pitz et al,2011[107]	USA, America	2000–2008	Blood samples	E-test GRD	2/167 (1.2)	
				PAP-AUC		
Musta et al, 2009[105]	USA, America	2002–2003	Blood samples	MHA	37/187 (19.8)	
				E-test	34/37 (93.0) SCC*mec* II	
					3/37 (7.0) SCC*mec* IV	
Sader et al, 2009[77]	USA, America	2002–2006	Blood samples	MET	36/268 (13.4)	
				PAP-AUC		
Pastagia et al, 2009[22]	USA, America	2002–2007	Blood samples	E-test	45/699 (6.4)	118/699 (16.9)
Khosrovaneh et al, 2004[47]	USA, America	2002/01–2002/12	Blood samples	BHI	3/22 (13.6)	
				PAP-AUC		
Casapao et al, 2014[44]	USA, America	2002/01–2013/06	All clinical samples	PAP-AUC	38/266 (18.8)	
					26/38 (68.4) SCC*mec* IV	
					11/38 (28.9) SCC*mec* II	
					1/38 (2.7) SCC*mec* III	
Khatib et al, 2011[92]	USA, America	2002–03 and 2005–06	Blood samples	MET	30/371 (8.1)	6/371 (1.6)
				PAP-AUC	26/30 (86.7) SCC*mec* II	6/6 (100) SCC*mec* II
					4/30 (13.3) others	
Rybak et al, 2008[21]	USA, America	2003–2007	All clinical samples	MET	76/917 (8.3)	3/917 (0.3)
				PAP-AUC	43/76 (56.9) SCC*mec* II	
					29/76 (38.4) SCC*mec* IV	
					4/76 (4.7) others	
Delgado et al, 2007[53]	Mexico, America	2003/09–2004/08	All clinical samples	PAP	1/152 (0.7)	
Musta et al, 2009[105]	USA, America	2005–2006	Blood samples	MHA	21/186 (11.3)	
				E-test	20/21 (93.0) SCC*mec* II	
					1/21 (7.0) SCC*mec* IV	
Kosowska-Shick et al, 2008[57]	USA, America	2006/08–2007/12	All clinical samples	MET	2/982 (0.2)	3/982 (0.3)
				PAP		2/3 (66.7) SCC*mec* II
						1/3 (33.3) SCC*mec* IV
Hafer et al, 2012[29]	USA, America	2007–2008	All clinical samples	MIC based	9/77 (11.7)	22/77 (28.6)
				PAP-AUC		11/22 (50.0) ST5
						11/22 (50.0) ST8
Fink et al, 2012[64]	USA, America	2008/02–2010/01	All clinical samples	E-test GRD	0/288 (0)	
				PAP-AUC		
Richter et al, 2011[35]	USA, America	2009/06–2009/12	All clinical samples	E-test GRD	11/4210 (0.4)	0/4210 (0)
				PAP-AUC		
Silveira et al, 2014[62]	Brazil, America	2009/03–2013/02	All clinical samples	E-test GRD	12/124 (9.7)	
				PAP-AUC		
Van Hal et al, 2011[37]	Australia, Oceania	1997–2008	Blood samples	PAP-AUC	54/465 (11.6)54/54 (100) ST239	
Charles et al, 2004[24]	Australia, Oceania	2001/07–2002/06	Blood samples	E-test	5/53 (9.4)	0/53 (0)
				PAP-AUC		
Horne et al, 2009[89]	Australia, Oceania	2005/03–2005/12	All clinical samples	MIC based	56/117 (47.9)	2/117 (1.7)
				PAP-AUC		

^a^ BHI: Brain Heart Infusion Agar; PAP: Population Analysis Profile; PAP-AUC: Population Analysis Profile–Area Under the Curve; MET: Macromethod E-test; MHA: Muller Hinton Agar; E-test GRD: E-test Glycopeptide Resistant Detection

**Table 2:** Prevalence of hVISA and VISA based on study period, origin of study, and isolate selection.^a^

	Category	Subcategory	No. Studies	No. Strains	Prevalence (%) (95% CI)
hVISA	Overall		76	99042	6.05 (4.78–7.48)
	Study period	Before 2006	42	40119	4.68 (3.19–6.41)
		2006–2009	10	6485	5.38 (2.40–9.48)
		2010–2014	5	680	7.01 (2.12–14.42)
	Continent	Asia	35	64692	6.81 (4.76–9.16)
		Europe-America	41	34350	5.60 (3.85–7.64)
	Clinical sample	Blood culture sample	21	5944	9.81 (6.71–13.42)
		All clinical sample	55	93098	4.68 (3.51–6.00)
VISA	Overall		38	68792	3.01 (1.62–4.83)
	Study period	Before 2006	20	13394	2.05 (0.95–3.55)
		2006–2009	4	5630	2.63 (0.29–7.22)
		2010–2014	2	2090	7.93 (0.06–26.67)
	Continent	Asia	18	55362	3.42 (1.10–6.99)
		Europe-America	20	13430	2.75 (1.19–4.91)
	Clinical sample	Blood culture samples	7	2542	2.00 (0.03–6.88)
		All clinical samples	31	66250	3.24 (1.67–5.29)

CI, confidence interval

^a^ References: [18–108].

### Prevalence of hVISA/VISA in different study periods

To analyze the trends in the changes in hVISA/VISA prevalence in recent years, we performed a subgroup analysis of the prevalence of these two types of strains according to the study year. Three periods (before 2006, 2006–2009, and 2010–2014) were designated. Some studies that did not conform to the periods (e.g., reported for 2003–2007) were not included in this analysis.

It can be seen from Table 2 that the prevalence of the hVISA isolates increased gradually from 4.68% (95% CI 3.19–6.41) of 40,119 MRSA strains before 2006 to 5.38% (95% CI 2.40–9.48) of 6485 strains in 2006–2009, reaching 7.01% (95% CI 2.12–14.42) of 680 strains in 2010–2014. The incidence of VISA was 2.05% (95% CI 0.95–3.55) of 13,394 strains before 2006, 2.63% (95% CI 0.29–7.22) of 5,630 strains in 2006–2009, and 7.93% (95% CI 0.06–26.67) of 2090 strains in 2010–2014. Thus, the frequency of VISA during the years 2010–2014 represents a 3.87-fold increase over the years before 2006.

### Prevalence of hVISA/VISA at different locations

The prevalence of hVISA/VISA differed among geographic regions in the subgroup analysis, as shown in Table 2. The prevalence of hVISA was 6.81% (95% CI 4.76–9.16) of 64,692 MRSA strains in 35 studies from Asia, and 5.60% (95% CI 3.85–7.64) of 34,350 strains in 41 studies from Europe/America. Moreover, 3.42% (95% CI 1.10–6.99) of 55,362 MRSA strains were VISA in 18 studies from Asia compared with 2.75% (95% CI 1.19–4.91) of 13,430 strains in 20 studies from Europe/America.

### Prevalence of hVISA/VISA in different clinical samples

In this subgroup analysis, we divided the MRSA strains into two groups. One group was isolated from only blood culture samples and the other from all clinical samples, including blood, sputum, pus, urine, and so forth (the authors of the original studies did not classify the prevalence rates in the different types of samples). In total, the frequency of hVISA was 9.81% (95% CI 6.71–13.42) in 5944 MRSA strains isolated from blood culture samples reported in 21 studies, significantly higher than in the group of all clinical samples (4.68% [95% CI 3.51–6.00] in 93,098 strains in 55 studies) (P = 0.023). The prevalence rates for VISA were 2.00% (95% CI 0.03–6.88) in 2542 blood-borne MRSA strains in seven studies, and 3.07% (95% CI 1.58–5.02) in 66,250 strains isolated from all clinical samples in another 32 studies (Table 2).

### Genetic backgrounds of hVISA/VISA

As shown in Table 3, 25 studies presented information on the genotypes of the hVISA/VISA strains. Sixteen studies that included 685 MRSA strains reported the staphylococcal cassette chromosome *mec* (SCC*mec*) types for hVISA. The predominant type was SCC*mec* II, which accounted for 48.16% of hVISA (95% CI 32.82–63.68), followed by SCC*mec* IV (18.07%; 95% CI 7.50–31.98) and SCC*mec* III (17.99%; 95% CI 7.69–31.42). SCC*mec* I accounted for only 2.12% (95% CI 0.70–4.30). Among the 454 strains from 10 studies that reported multilocus sequence typing (MLST), 11 ST types were identified. ST239 was found in 58.62% (95% CI 22.98–89.73) of hVISA, followed by ST5 in 14.45% (95% CI 4.59–28.53) and ST72 in 3.28% (95% CI 0.98–6.88). The SCC*mec* types in VISA strains were reported in nine studies, which included 97 strains. SCC*mec* II was predominant (37.74%, 95% CI 10.01–70.94), followed by SCC*mec* III (32.72%, 95% CI 3.35–73.85). SCC*mec* I and SCC*mec* IV accounted for 11.79% (95% CI 0.01–40.76) and 10.08% (95% CI 1.77–24.05) of isolates, respectively. Six ST types were reported among the VISA strains in 62 strains in six studies. The most prevalent ST types were ST239 (27.05%, 95% CI 2.34–65.22) and ST5 (22.77%, 95% CI 4.66–49.26) (Table 3).

**Table 3:** Genetic prevalence of hVISA and VISA.^a^

	Category	Subcategory	No. Studies	No. Strains	Prevalence (%) (95% CI)
hVISA	SCC*mec*		16	685	
		SCC*mec* I			2.12 (0.70–4.30)
		SCC*mec* II			48.16 (32.82–63.68)
		SCC*mec* III			17.99 (7.69–31.42)
		SCC*mec* IV			18.07 (7.50–31.98)
	MLST		10	454	
		ST239			58.62 (22.98–89.73)
		ST5			14.45 (4.59–28.53)
		ST72			3.28 (0.98–6.88)
		ST59			1.64 (0.28–4.10)
		ST900			0.95 (0.13–2.49)
		Others (ST1, ST247, ST228, ST398, ST45, ST1301)			9.51 (0.48–27.95)
VISA	SCC*mec*		9	97	
		SCC*mec* I			11.79 (0.01–40.76)
		SCC*mec* II			37.74 (10.01–70.94)
		SCC*mec* III			32.72 (3.35–73.85)
		SCC*mec* IV			10.08 (1.77–24.05)
	MLST		6	62	
		ST239			27.05 (2.34–65.22)
		ST5			22.77 (4.66–49.26)
		Others (ST59, ST72, ST228, ST8)			42.44 (10.44–78.65)

CI, confidence interval

^a^References: [21, 26, 28, 29, 33, 44, 46, 52, 55, 59, 65, 70–72, 74, 76, 83, 84, 86, 92, 105]

## Discussion

The infections caused by MRSA are problematic because they entail high mortality and only limited antimicrobial drugs are available for their treatment [109]. Vancomycin has generally been the first drug of choice for the treatment of MRSA infections [110]. However, studies have reported that the treatment failure rate for vancomycin is increasing. Takesue et al. studied 128 strains of MRSA causing bacteremia and reported that the efficacy of vancomycin in patients infected with strains with a vancomycin MIC of ≤ 1 μg/ml was 78.8%, whereas it was only 30.0% for patients infected with strains with an MIC of 2 μg/ml [111]. Moore et al. also investigated MRSA bacteremia, and defined treatment failure as a composite of mortality, microbiological failure, and/or the recurrence of infection. The treatment failure rate was 31% in patients infected with 118 MRSA strains with vancomycin MIC > 1 μg/ml [112]. Casapao et al. defined treatment failure as bacteremia for > 7 days or death attributable to MRSA, and observed 64.4% treatment failure in 266 patients with MRSA endocarditis [44]. hVISA and VISA are thought to be among the primary causes of treatment failure. However, in 76 studies (including 99,042 strains) chosen for our analysis, the prevalence of hVISA was only 6.05%, and the prevalence of VISA was only 3.01% in 68,792 strains in 38 studies. Therefore, we speculate that the incidence of hVISA/VISA was underestimated, possibly because of the resistance mechanisms and biological characteristics of these strains. Unlike MRSA and vancomycin-resistant *S*. *aureus* (VRSA), the genetic backgrounds associated with hVISA/VISA remain unclear, and a molecular biological method to detect these strains is not yet available. The growth rates of hVISA/VISA are also slow [113, 114]; hence, conventional methods, such as the Kirby–Bauer and instrument-based methods, do not produce accurate results. The PAP-AUC method is considered the gold standard technique for detecting hVISA. However, this method is time-consuming, cumbersome, and unsuitable for clinical laboratories [69], so a significant number of strains may have been missed. Therefore, there is an urgent need for a convenient and effective method with which to detect these strains.

To analyze the trends in the prevalence of hVISA/VISA in recent years, we divided the study period into three periods: before 2006, 2006–2009, and 2010–2014. The first period used the initial resistance breakpoint (vancomycin MIC of 8–16 μg/ml) and the two later periods used the present resistance breakpoint (vancomycin MIC of 4–8 μg/ml). Our study suggests that the prevalence of hVISA/VISA has been increasing in recent years. We consider that the more frequent use of vancomycin for MRSA infections is responsible for this situation because the high prevalence of hVISA/VISA reflects the level of vancomycin use [115, 116]. The inappropriate management of drug-resistant strains has accelerated the spread of hVISA/VISA, and the change in the vancomycin-resistance breakpoint has also contributed to the increase in the prevalence rate.

Since the first reports of hVISA/VISA, the occurrence rates of these strains have varied throughout the world: the incidence of hVISA was 6.81% in Asia and 5.60% in Europe/America, and that of VISA was 3.42% and 2.75%, respectively. Current evidence supports the proposition that hVISA/VISA is more endemic in Asian countries than in Europe/America. Several factors may account for this situation. First, most countries in Europe and America are developed, with high public hygiene standards and scrupulous antimicrobial treatments [69, 101, 102]. Second, the control of nosocomial infections is more successful in European and American countries [41, 95]. Third, Asia is the most populous region of the world, which can create an environment amenable to microbial transmission. The pooled prevalence rate for hVISA in mainland China was 15.78% [58, 71, 83, 84], and in India, the pooled prevalence rates for of hVISA and VISA were 12.41% and 15.09%, respectively [40, 51, 52, 85, 99]. Fourth, because far more MRSA infections occur in Asian countries [117], vancomycin has been used more frequently for their treatment. Therefore, it is not surprising that hVISA and VISA are more common in Asia than elsewhere.

Previous studies have demonstrated that hVISA/VISA are prevalent among bacteremic specimens, and that these strains can persist in the bloodstream for a long time [19]. Our analysis confirms that hVISA is more common in blood-borne MRSAs, consistent with previous opinion. However, the prevalence of VISA was not obviously higher among isolates from blood culture samples than other samples. The reason for this is unclear, but this result suggests that not only blood culture samples but all clinical samples should be given attention.

The *mecA* gene, which is located within the SCC*mec* element, is the specific genetic mechanism of methicillin resistance [118]. Many epidemiological studies have demonstrated that community-associated MRSA (CA-MRSA) can be distinguished from hospital-acquired MRSA (HA-MRSA) by the type of the SCC*mec* element present. The most common SCC*mec* types in CA-MRSA strains are SCC*mec* IV and V, whereas SCC*mec* I, II, and III predominate in HA-MRSA strains [119]. The results of our pooled analysis show that SCC*mec* II and III were the most prevalent molecular types among the VISA strains. Previous studies have demonstrated limited vancomycin-resistance potential in SCC*mec* IV MRSA clones [120]. However, we found that the prevalence of SCC*mec* IV in hVISA was similar to that of SCC*mec* III. This phenomenon suggests that hVISA is not limited to typical “hospital” clones of *S*. *aureus*.

MLST is a powerful and highly discriminatory method for analyzing the population structures and epidemiology of *S*. *aureus* [121]. Our study demonstrates that ST239 and ST5 are the most epidemic genotypes of hVISA/VISA. ST239 and ST5 are two international HA-MRSA lineages prevalent in Asia, South America, and Eastern Europe [122, 123]. ST239 MRSA strains are typically resistant to many classes of antibiotics, including β-lactams, fluoroquinolones, aminoglycosides, and macrolide antibiotics. Our results strongly suggest that hVISA and VISA are highly prevalent among international epidemic MRSA strains. Moreover, the genetic backgrounds of these strains are complex, and many ST types are dispersed among hVISA/VISA isolates, including ST59, ST72, and ST900 [70, 72, 74].

The present study had several limitations. Genetic information was available in only 27% (25/91) of the studies we reviewed, which could have led to publication bias and influenced our results. There was also considerable heterogeneity between studies because they differed in various study variables, such as the patient populations examined, testing methodologies used, study durations, previous vancomycin therapy, and concomitant illnesses. These confounding factors could not be circumvented with subgroup analyses. As in previous meta-analyses in which unexplained heterogeneity was identified, we accommodated this condition by using REM, in which the effects underlying the results of different studies are assumed to be drawn from a normal distribution [124]. However, this heterogeneity could not be balanced out by REM alone, so that the stability of the final results must have been affected by the heterogeneity of the sample.

In summary, the results of our study suggest that the prevalence rates of hVISA/VISA have increased in recent years. Our data also supports the view that hVISA/VISA are more prevalent in Asian countries than in Europe/America. Our study confirms that hVISA strains are more common in blood-borne MRSA than in other MRSA. Finally, the most epidemic genotypes of hVISA/VISA are SCC*mec* II and SCC*mec* III on SCC*mec* typing, and ST239 and ST5 on MLST typing, which are predominant among the HA-MRSA strains. However, the incidence of hVISA/VISA is grossly underestimated. Therefore, the detection of hVISA/VISA must be strengthened, especially in samples from patients with bacteremic HA-MRSA infections, and the use of vancomycin and nosocomial infections must be urgently and strictly controlled, particularly in Asian hospitals.

## Supporting Information

S1 PRISMA Checklist PRISMA 2009 checklist. (DOC)
